# Assessing the Clinical Robustness of Digital Health Startups: Cross-sectional Observational Analysis

**DOI:** 10.2196/37677

**Published:** 2022-06-20

**Authors:** Sean Day, Veeraj Shah, Sari Kaganoff, Shannon Powelson, Simon C Mathews

**Affiliations:** 1 Rock Health Inc San Francisco, CA United States; 2 Department of Public Health and Primary Care University of Cambridge Cambridge United Kingdom; 3 Whiting School of Engineering Johns Hopkins University Baltimore, MD United States; 4 Division of Gastroenterology & Hepatology Johns Hopkins University School of Medicine Baltimore, MD United States

**Keywords:** digital health, health tech, software as a medical device (SaaMD), real-world evidence, venture capital

## Abstract

**Background:**

The digital health sector has experienced rapid growth over the past decade. However, health care technology stakeholders lack a comprehensive understanding of clinical robustness and claims across the industry.

**Objective:**

This analysis aimed to examine the clinical robustness and public claims made by digital health companies.

**Methods:**

A cross-sectional observational analysis was conducted using company data from the Rock Health Digital Health Venture Funding Database, the US Food and Drug Administration, and the US National Library of Medicine. Companies were included if they sell products targeting the prevention, diagnosis, or treatment phases of the care continuum. Clinical robustness was defined using regulatory filings and clinical trials completed by each company. Public claims data included clinical, economic, and engagement claims regarding product outcomes made by each company on its website.

**Results:**

A total of 224 digital health companies with an average age of 7.7 years were included in our cohort. Average clinical robustness was 2.5 (1.8 clinical trials and 0.8 regulatory filings) with a median score of 1. Ninety-eight (44%) companies had a clinical robustness score of 0, while 45 (20%) companies had a clinical robustness score of 5 or more. The average number of public claims was 1.3 (0.5 clinical, 0.4 economic, and 0.4 engagement); the median number of claims was 1. No correlation was observed between clinical robustness and number of clinical claims (*r*^2^=0.02), clinical robustness and total funding (*r*^2^=0.08), or clinical robustness and company age (*r*^2^=0.18).

**Conclusions:**

Many digital health companies have a low level of clinical robustness and do not make many claims as measured by regulatory filings, clinical trials, and public data shared online. Companies and customers may benefit from investing in greater clinical validation efforts.

## Introduction

The digital health sector has grown rapidly over the past decade [1,2]. There are more than 1900 digital health startup companies in the United States that have raised more than US $2 million in venture funding, which in total have raised more than US $77 billion in venture capital funding since 2011 [3].

Although growth is apparent, the ability to measure impact is not. Several studies have highlighted the need for greater clinical validation [4,5] and found that many solutions were not supported by robust clinical evidence [6,7] and demonstrated mixed results on cost savings and cost-effectiveness [8,9]. In addition, there is evidence that some claims made by digital health companies have been misleading [10-12], with a few highly publicized cases resulting in legal action by the Federal Trade Commission and state attorneys general [13-15]. Most studies focusing on clinical impact are narrowly defined to specific clinical therapeutic areas (eg, diabetes, cardiac arrhythmia), making it difficult to extrapolate findings to the broader field of digital health [16-22]. Additional limitations include a small sample of companies and the use of publications as a proxy for clinical impact. Other studies in digital health have examined larger clinical trends, such as growth in clinical trials, but these often lack data on clinical and customer focus [23,24].

As a result, the literature is often too narrow or too broad to enable an understanding of clinical impact. In addition, no studies have examined both clinical rigor and public claims made by companies. To address these limitations, we sought to comprehensively examine the topic of clinical robustness in digital health companies by using a more comprehensive definition of clinical rigor and examining companies’ public claims across the most in-depth database of US-based digital health companies. These findings provide additional context for all stakeholders in health technology that rely on a more accurate characterization of digital health solutions.

## Methods

### Population

Companies were identified using the Digital Health Venture Funding Database maintained by Rock Health Inc, a digital health venture fund and advisory firm, which has been used in prior studies [25-27]. The database includes all digital health companies with headquarters in the United States that have raised at least one venture funding round of US $2 million or more since 2011. Our analysis included companies that sell products targeting the prevention, diagnosis, or treatment phases of the care continuum, which raised at least one round of funding between 2011 and 2020. Digital health companies are defined as those that build and sell digital technologies in health care [28,29].

### Company Variables

Total venture funding, clinical area(s) of focus, care continuum phase(s), and customer data were collected for each company. Clinical areas represented 1 of 20 specific clinical domains (eg, cardiovascular, nephrology). Care continuum phase was defined according to the following: prevention, diagnosis, or treatment. Customer type referred to the category of buyer for a company’s products, such as payer (ie, health insurance companies), biopharma (ie, pharmaceutical or biopharmaceutical companies), and medical devices (ie, companies that manufacture medical devices). Companies can be categorized into multiple categories (ie, companies may address multiple phases of the care continuum or multiple clinical areas). Company variables were gathered from the Digital Health Venture Funding Database, which Rock Health maintains using publicly available information such as company websites, press releases, and US Securities and Exchange Commission filings. Data for companies were collected through August 3, 2021.

### Claims Variables

The number and type of claims, defined as unique quantitative statements about product outcomes, made on a company’s website were collected in the following categories: clinical, economic, and engagement. The definitions of these claim types are detailed in Table 1. Data were obtained by reviewing all pages of a company’s website, excluding links to external pages such as press releases, between May 3, 2021, and August 3, 2021.

**Table 1:** Types of claims made by digital health companies.

Claim type	Definition	Claim subtype
Engagement	A quantitative statement on how engaged users are with the technology or that it provides a better patient experience	Number of active users/user retention rate Measure of user engagement per unit time (ie, monthly, annual)
Economic	A quantitative statement about a product’s impact on health-related expenses or revenue for the buyer or end user of the product	Money saved per stakeholder, including return on investment (either to patient, payer, or provider) or as compared to competition/existing standard of care New revenue generation for stakeholders Decrease in health care services utilization
Clinical	A quantitative statement about a product’s impact on patient health or well-being	Diagnostic efficacy General clinical improvement/reduction in symptoms or condition Change in objective clinical metric (including validated patient-reported metrics) Disease cure (reversal or permanent cure of a disease) Prevention (prevents progression or occurrence of a specific disease) Improvement in quality of life Improvement in medication adherence

### Clinical Robustness Variables

We collected regulatory data from the Food and Drug Administration (FDA), including the number of 510(k), De Novo, and premarket approval filings (where the company was listed as the “Requester” on fda.gov). In addition, we collected the number and type of clinical trials by searching ClinicalTrials.gov where the company was listed as “Sponsor / Collaborator.” Data on both FDA filings and clinical trials were collected between July 1, 2021, and September 2, 2021, through a combination of web scraping and manual searching. Data collection on claims was completed by at least 3 authors, with blinded cross-review to ensure consistency in data collection.

A “clinical robustness” score was calculated for each company, defined as the sum of the number of regulatory filings and clinical trials. Each regulatory filing and clinical trial was weighted equally in the calculation.

### Data and Statistical Analysis

All data were stored in Microsoft Excel (Microsoft Corp), where all descriptive and statistical analyses including coefficient of correlation between variables were calculated.

## Results

### Population Characteristics

There were 224 companies in the cohort, with an average age of 7.7 years. Collectively, these companies have raised a total of US $8.2 billion in venture capital funding since 2011. The companies spanned 3 phases of the care continuum, with 25 offering solutions for prevention, 106 offering solutions for diagnosis, and 110 companies offering solutions for treatment (Multimedia Appendix 1). Some companies offered products across multiple phases of the care continuum and were therefore counted in multiple categories. Average company funding was similar across phases of care—prevention companies raised US $35.3 million; diagnosis companies raised US $37.8 million; and treatment companies raised US $37.9 million.

### Clinical Robustness

The average clinical robustness score for all companies was 2.5 (clinical trials: 1.8; regulatory filings: 0.8). The median clinical robustness score was 1, with 98 companies (44%) having a score of 0 and 34 companies (15%) having a score of 1. Diagnosis companies had the highest average clinical robustness scores (2.8), followed by treatment companies (2.2), and then prevention companies (1.9).

The average clinical robustness score of companies that sold to employers was 3.1, compared to 2.0, 2.2, and 2.7 for those companies that sold to payers, consumers, and providers, respectively (Multimedia Appendix 2). Fifteen of the 18 clinical areas had a higher average number of clinical trials than regulatory filings. The distribution of companies by clinical robustness score can be found in Table 2.

**Table 2:** Distribution of companies by clinical robustness score.

Clinical robustness score	Companies, n (%)
0	98 (44)
1	34 (15)
2	24 (11)
3	15 (7)
4	8 (4)
5	13 (6)
6	7 (3)
7	4 (2)
8	7 (3)
9	1 (0)
≥10	13 (6)

### Claims

**Figure 1:**
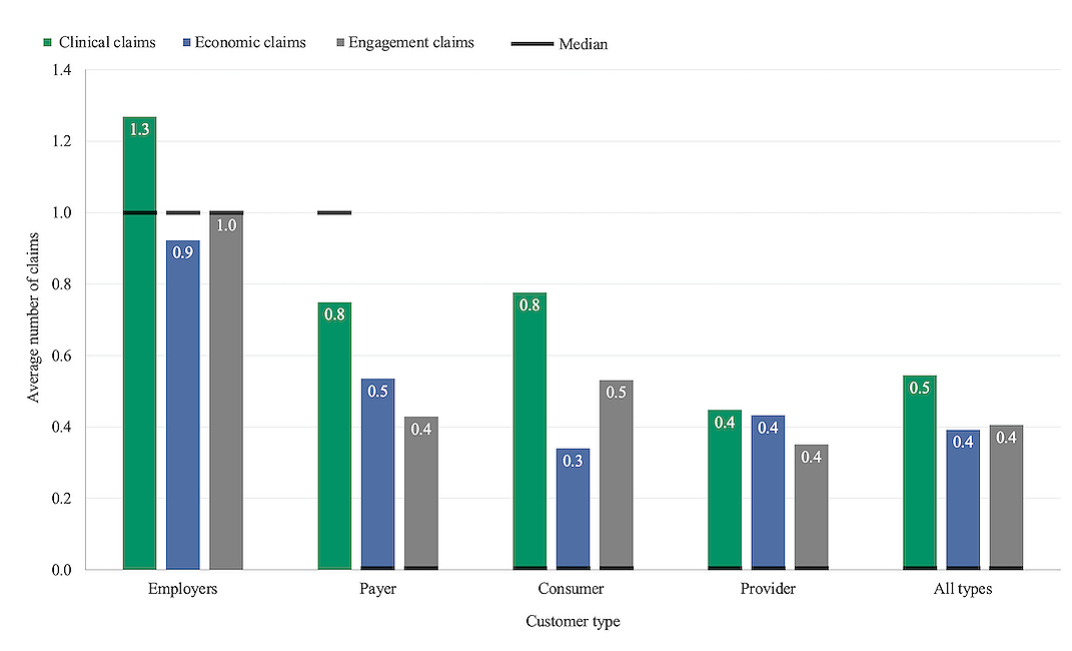
Average number of claims made by start-ups broken out by customer type.

The average number of total claims for all companies was 1.3 (0.5 clinical, 0.4 economic, and 0.4 engagement) with the median number of total claims equal to 1 (0 clinical, 0 economic, 0 engagement) (Multimedia Appendix 3). The median number of claims of any type was zero for companies that sold to consumers and providers. The median number of economic and engagement claims for companies that sold to payers was also zero. Companies that sold to employers made more clinical, economic, and engagement claims than companies that sold to all other customer types (Figure 1). Many companies (43%) made zero claims.

### Clinical Robustness and Claims

There was no correlation between clinical robustness and number of clinical claims (*r*^2^=0.02), clinical robustness and total funding (*r*^2^=0.08), or clinical robustness and company age (*r*^2^=0.18). In addition, there was no strong correlation between clinical areas that had higher clinical robustness scores and clinical areas where companies have received higher average funding (*r*^2^=0.07).

## Discussion

### Principal Findings

**Figure 2:**
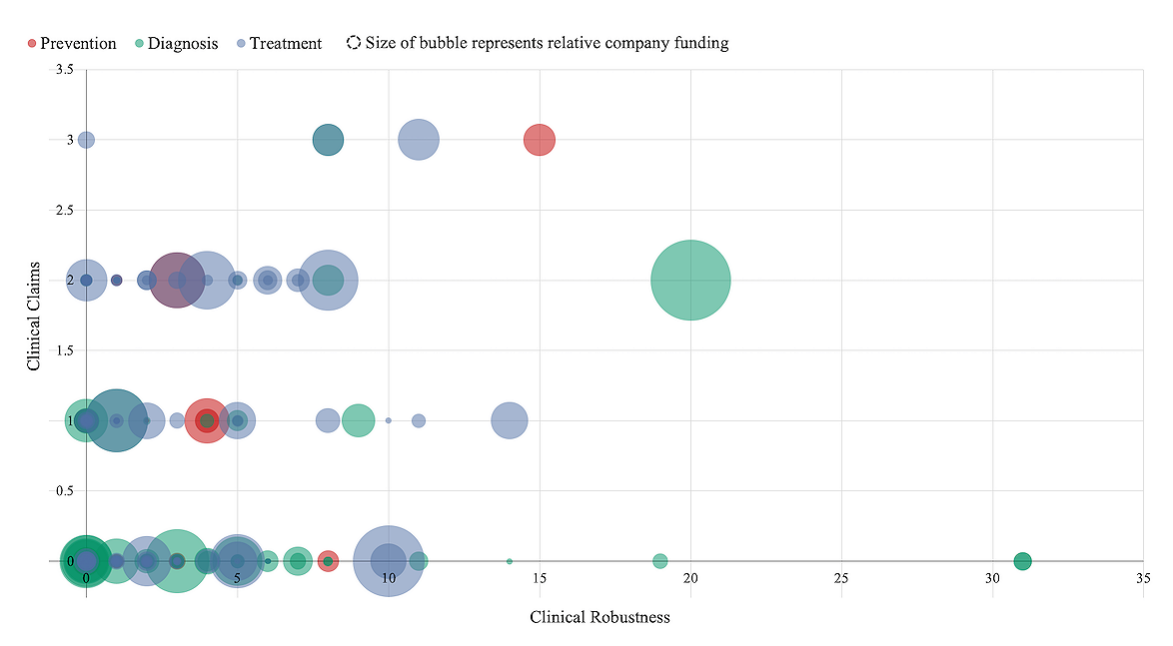
Clinical robustness, total claims, and total company funding across the digital health landscape.

Our findings indicate that many venture-backed startups in digital health have limited clinical robustness as measured by regulatory filings and clinical trials. There was, however, a sizable minority (20%) that had a score of 5 or more, suggesting a small population of rigorously tested solutions (Table 2). Although this subpopulation may portend progress, the lack of meaningful clinical validation for nearly half of digital health companies (44% had a clinical robustness score of 0) highlighted a major gap in health care technology today. The lack of overall correlation between a company’s total venture funding and its clinical robustness score similarly highlighted a significant asymmetry in how companies are potentially valued in today’s marketplace (ie, no correlation between clinical impact and funding) (Figure 2). However, it is possible that funding amounts reflect future anticipated value rather than current value [30].

**Figure 3:**
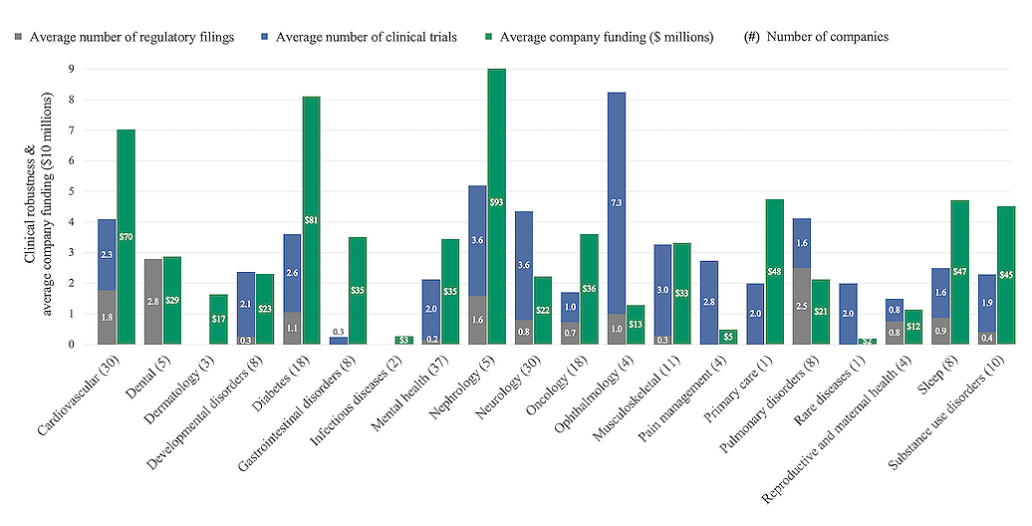
Funding and clinical robustness across various clinical areas.

Although average clinical robustness was quite low across the population, there was significant variation across clinical areas (eg, high in cardiovascular and nephrology and low in oncology and primary care) (Figure 3). This may reflect the varying levels of technological maturity of digital health solutions across clinical disciplines. Prior literature points to significant differences in technological maturity between well-funded clinical areas such as diabetes [27] and less well-funded areas such as reproductive and maternal health [31].

The average and median number of all claims was also low. These findings indicated that digital health companies largely did not share outcomes publicly. This may have reflected a desire to keep this data private, but more likely represented a lack of meaningful analyses of any impact (clinical, economic, or engagement) since this data could be used as a competitive differentiator if shared publicly. Separately, we identified 32 companies that had one or more clinical claims and a clinical robustness score of 0. These findings may suggest a disconnect between marketing and evidence. Additional future research could examine the links between public claims and clinical trials or regulatory filings (ie, are individual claims directly supported by clinical trials or regulatory filings?).

Companies that sold to employers made more claims and had higher clinical robustness scores compared to other customer types (Figure 1). This may suggest that this customer base is currently the most competitive and/or has the highest entry standards, despite accounting for a relatively small percentage (<12%) of companies in the sample. A competitive employer market for digital health tools is unsurprising given the rising cost burden of health care on employers [32] and evidence that employers are increasingly offering digital health benefits to improve health outcomes [33,34] and contain health care costs [35,36].

### Limitations

Our definition of clinical robustness was limited to clinical trials and regulatory findings, which remain proxies for effectiveness and require companies to register their activities. However, we believe these elements offer a better estimation of clinical rigor than publications, which can lag in timing and may not always relate to clinical outcomes. We chose to focus on clinical trials and regulatory filings because they were publicly available data and are generally undertaken to demonstrate an impact on clinical outcomes. Our approach to measuring clinical robustness equally accounts for clinical trials that demonstrate a technology does and does not work. Additionally, our data collection methodology could have missed clinical trials that were not registered on ClinicalTrials.gov or regulatory filings that were submitted to the FDA under an individual’s name rather than the company name (both scenarios are atypical). Future research could incorporate condition- or disease-specific metrics of effectiveness that are then standardized across clinical areas to provide a more accurate measure of clinical impact.

In addition, our cohort only included venture-backed companies above US $2 million in funding. This may have resulted in selection bias by excluding both ends of the spectrum (ie, not including earlier stage companies or large conglomerate technology companies). Although our population excludes these companies, we believe that our sample represents the most comprehensive assessment of outcomes or claims across the digital health landscape and encompasses the majority of funded companies and activity in this space. As a point of reference, the average seed stage deal size in 2021 was US $3.5 million, 75% higher than our minimum funding threshold [37].

### Conclusions

Despite the hundreds of digital health companies targeting the myriad of needs across the care continuum, clinical robustness and public communication of claims remains low across much of the sector. These results highlight a significant opportunity for companies to differentiate themselves and for customers to demand greater validation for the products and services they purchase.

## Appendix

Multimedia Appendix 1 Digital health company characteristics across the care continuum, clinical areas, and customer types.

Multimedia Appendix 2 Clinical robustness scores, clinical trials, and regulatory filings.

Multimedia Appendix 3 Claims made by digital health companies.

## Abbreviations

FDA: Food and Drug Administration
