# Supplementary material for: Assessing the Clinical Robustness of Digital Health Startups: Cross-sectional Observational Analysis
Source: J Med Internet Res. 2022 Jun 20;24(6):e37677. doi: 10.2196/37677 (PMC9253972; doi:10.2196/37677)
Supplement: Multimedia Appendix 1 [file jmir_v24i6e37677_app1.pdf]

		Number of companies	Average funding (in millions of US $)	Years since founding
All companies		224	36.6	7.7
**Care continuum phase**				
	Prevention	25	35.3	8.7
	Diagnosis	106	37.8	8.4
	Treatment	110	37.9	6.8
**Clinical area**				
	Cardiovascular	30	70.3	9.4
	Dental	5	28.8	5.8
	Dermatology	3	16.5	6.3
	Developmental disorders	8	23.1	6.9
	Diabetes	18	81.1	9.7
	Gastrointestinal disorders	8	35.2	6.1
	Infectious diseases	2	2.8	3.0
	Mental health	37	34.5	7.0
	Nephrology	5	92.7	9.6
	Neurology	30	22.3	9.0
	Oncology	18	36.2	7.8
	Ophthalmology	4	13.0	10.0
	Musculoskeletal	11	33.3	6.3
	Pain management	4	4.9	4.5
	Primary care	1	47.5	8.0
	Pulmonary disorders	8	21.3	11.6
	Rare diseases	1	2.0	10.0
	Reproductive and maternal health	4	11.5	8.3
	Sleep	8	47.3	7.6
	Substance use disorders	10	45.2	6.2
**Customer type**				
	Individual consumers	94	27.8	6.7
	Biopharma	12	42.9	7.4
	Employers	26	57.9	7.9
	Providers	134	39.8	8.4
	Payers	28	65.0	6.6
	Medical devices	3	19.9	9.3
	Pharmacies	3	16.3	5.0
	Alternative health care providers	2	8.2	7.0
