# Supplementary material for: Assessing the Clinical Robustness of Digital Health Startups: Cross-sectional Observational Analysis
Source: J Med Internet Res. 2022 Jun 20;24(6):e37677. doi: 10.2196/37677 (PMC9253972; doi:10.2196/37677)
Supplement: Multimedia Appendix 2 [file jmir_v24i6e37677_app2.pdf]

	Average clinical robustness score	Average clinical trials	Average regulatory filings (all types)	Average de novo filings	Average PMA[a] filings	Average 510(k) filings
All companies	2.5	1.8	0.8	0.1	0.0	0.7
**Care continuum phase**						
Prevention	1.9	1.6	0.3	0.0	0.0	0.3
Diagnosis	2.8	1.9	1.0	0.1	0.0	0.9
Treatment	2.2	1.6	0.6	0.0	0.0	0.5
**Clinical area**						
Cardiovascular	4.1	2.3	1.8	0.1	0.0	1.7
Dental	2.8	0.0	2.8	0.0	0.0	2.8
Dermatology	0.0	0.0	0.0	0.0	0.0	0.0
Developmental disorders	2.4	2.1	0.3	0.3	0.0	0.0
Diabetes	3.6	2.6	1.1	0.1	0.0	1.0
Gastrointestinal disorders	0.3	0.3	0.0	0.0	0.0	0.0
Infectious diseases	0.0	0.0	0.0	0.0	0.0	0.0
Mental health	2.1	2.0	0.2	0.0	0.0	0.1
Nephrology	5.2	3.6	1.6	0.0	0.0	1.6
Neurology	4.4	3.6	0.8	0.2	0.0	0.6
Oncology	1.7	1.0	0.7	0.0	0.1	0.7
Ophthalmology	8.3	7.3	1.0	0.0	0.0	1.0
Musculoskeletal	3.3	3.0	0.3	0.0	0.0	0.3
Pain management	2.8	2.8	0.0	0.0	0.0	0.0
Primary care	2.0	2.0	0.0	0.0	0.0	0.0
Pulmonary disorders	4.1	1.6	2.5	0.0	0.0	2.5
Rare diseases	2.0	2.0	0.0	0.0	0.0	0.0
Reproductive and maternal health	1.5	0.8	0.8	0.0	0.0	0.8
Sleep	2.5	1.6	0.9	0.1	0.0	0.8
Substance use disorders	2.3	1.9	0.4	0.1	0.0	0.3
**Customer type**						
Individual consumers	2.2	1.8	0.4	0.0	0.0	0.4
Biopharma	1.0	0.9	0.1	0.0	0.0	0.1
Employers	3.1	2.8	0.3	0.0	0.0	0.2
Providers	2.7	1.6	1.1	0.1	0.0	1.0
Payers	2.0	1.6	0.4	0.0	0.0	0.3
Medical devices	0.0	0.0	0.0	0.0	0.0	0.0
Pharmacies	1.0	0.0	1.0	0.3	0.0	0.7
Alternative health care providers	0.5	0.5	0.0	0.0	0.0	0.0

[a]PMA: premarket approval.
